# BDNF Val66Met Polymorphism on Functional MRI During n-Back Working Memory Tasks

**DOI:** 10.1097/MD.0000000000001586

**Published:** 2015-10-23

**Authors:** Chih-Chung Chen, Chi-Jen Chen, Dean Wu, Nai-Fang Chi, Po-Chih Chen, Yen-Peng Liao, Hung-Wen Chiu, Chaur-Jong Hu

**Affiliations:** From the Graduate Institute of Biomedical Informatics, Taipei Medical University, Taipei (CCC, HWC); Department of Neurology (CCC, DW, NFC, PCC, CJH); Department of Radiology, Taipei Medical University-Shuang Ho Hospital, New Taipei City, Taiwan (CJC, YPL); Human Brain Research Center, Kyoto University Graduate School of Medicine, Kyoto, Japan (YPL); Department of Neurology, Medical School, Taipei Medical University, Taipei (CJH); and Brain and Consciousness Research Center, Shuang Ho Hospital, New Taipei City, Taiwan (CJH).

## Abstract

Val66Met polymorphism on the brain-derived neurotrophic factor (*BDNF*) gene is associated with hippocampal pathology and impaired episodic memory. However, the influence of this polymorphism on working memory (WM) performance and patterns of brain activation is controversial. This study investigated the effects of BDNF Val66Met polymorphism on functional magnetic resonance imaging (fMRI) during n-back WM tasks in healthy middle-aged adults.

A total of 110 participants without subjective or objective cognitive impairment underwent BDNF genotyping. Eleven Met allele carriers and 9 noncarriers underwent fMRI during WM tasks.

The WM performance was similar between the 2 groups. Increased brain activation in response to increases in WM loads was observed in both groups. The Met allele carrier group showed consistently lower brain activation in the right superior frontal gyrus (SFG) and the middle occipital gyrus than that of the noncarrier group (*P* < 0.001). No brain region showed increased activation during WM tasks in the Met allele group.

BDNF Val66Met polymorphism may affect the WM network. Met allele carriers have lower brain activation in the right SFG and middle occipital gyrus than do noncarriers during WM tasks. Defective development of the WM network during brain maturation or differentiation is a possible mechanism. Additional studies with a larger sample and longer follow-up period are warranted.

## INTRODUCTION

Brain-derived neurotrophic factor (BDNF) is widely distributed in the central nervous system. BDNF and its receptor tropomyosin receptor kinase B are highly expressed in the hippocampus, hypothalamus, and cortex.^1^ BDNF participates in activity-dependent plasticity processes, such as long-term potentiation, learning, and memory.^2^ Reduction in hippocampal BDNF expression has been associated with normal aging and age-related memory decline.^3^ A common single-nucleotide polymorphism on the *BDNF* gene, rs6265, results in an amino acid change from valine to methionine at codon 66 (Val66Met) in the prodomain of BDNF. This polymorphism influences intracellular BDNF trafficking and secretory regulation.

BDNF Val66Met polymorphism also affects memory function and motor learning and has been linked to several neuropsychiatric disorders.^4,5^ Several studies have demonstrated an association between BDNF Val66Met and impaired cognitive function in the memory and nonmemory domains.^6–11^ The polymorphism is also closely related to hippocampal pathology. Egan et al demonstrated that hippocampal *N*-acetyl aspartate levels in Met allele carriers decreased in a genetic and dose-dependent manner by using magnetic resonance spectroscopic imaging.^6^ Bueller et al showed that the Met allele was associated with an 11% reduction in the volume of hippocampal formation.^12^ In addition, a meta-analysis in 2012 showed lower total hippocampal volumes in Met allele carriers.^13^ Although most studies on BDNF Val66Met polymorphism demonstrated that Met allele carriers have a higher cognitive deficit risk, studies have also documented greater cognitive impairment in Met allele noncarriers.^14,15^

Working memory (WM) capacity can be measured using an n-back task in functional magnetic resonance imaging (fMRI). The participant monitors a series of stimuli and responds when a stimulus presented is same as that presented n trials previously. Val66Met polymorphism psychometrically and physiologically affects the hippocampus-dependent episodic memory.^6,16–18^ However, studies on the Met allele and WM performance in healthy participants have yielded contradictory results. Two studies have revealed poor WM performance in Met allele carriers.^9–11^ However, several studies have revealed no considerable difference between carriers and noncarriers.^6,10,19,20^ In addition, a previous fMRI study demonstrated disrupted hippocampal deactivation in Met allele carriers performing n-back WM tasks.^6^ A recent study showed significant overactivation of the right dorsolateral prefrontal cortex and the superior parietal lobule in healthy Met allele carriers under a high (2-back > 0-back) WM load, even though their WM performance was noninferior.^20^ The authors proposed that Met allele carriers have a compensatory mechanism for maintaining normal task performance; however, these results have not been reproduced in other experiments. In the present study, we compared changes of brain activation under low and high WM loads between healthy middle-aged Met allele carriers and noncarriers.

## MATERIALS AND METHODS

### Participants

A total of 110 people who underwent physical examination at Taipei Medical University-Shuang Ho Hospital participated in this study. All participants provided written informed consent, and the study was approved by the Taipei Medical University Joint Institutional Review Board. The participants completed the eight-item informant interview to differentiate aging and dementia (AD8) questionnaire^21^ and were assessed using the mini-mental status examination^22^ (MMSE) for subjective and objective cognitive impairment. People with a history of psychiatric disorders and an AD8 score > 2 or MMSE score < 26 were excluded. Therefore, people without cognitive impairment were selected for further genetic analysis, and 85 Met allele carriers and 25 noncarriers were identified. Eleven participants among the Met allele carriers (9 heterozygotes and 2 homozygotes) underwent fMRI analysis. Nine age- and sex-matched participants were recruited from the sample of noncarriers.

### Genotyping

Genomic DNA was extracted using the Blood Genomic DNA Extraction Midiprep System. DNA was amplified using PCR in a thermal cycler with a BDNF forward primer, 5′-GCTGACACTTTCGAACAT-3′, and reverse primer, 5′-ACCCTCATGGACAAGTTT-3′. The PCR mixture contained 1 μg of DNA, 100 ng of primers, 50 μM each dNTP, 1× PCR buffer, and 2.5 units of Taq polymerase. The reactions were carried out through 35 cycles of denaturation for 1.5 minutes at 94 °C, annealing for 1.5 minutes, and extension for 2.5 minutes at 72 °C. After PCR amplification, 5 units of restriction enzyme, NIaII, were added to each reaction mixture and incubated overnight at 37 °C in a water bath to differentiate Val66Met polymorphism.^23^ Electrophoresis of the digested products was conducted on a 4% agarose gel stained with ethidium bromide. Gel images were collected using an image system, and the digestion fragment size was evaluated through comparison with size marker.

### Working Memory Task

The n-back WM protocol applied in our previous study.^24^ Before the fMRI scan, we explained the procedure of the task to all participants. In order to reduce anxiety, each of them was supervised to practice the task before entering the scanner room. We designed 3 conditions in the n-back tasks – 0-, 1-, and 2-back. In 0-back condition, the participants decided whether the number shown currently matched the predetermined number assigned by the examiner. This condition used a minimal WM load and the functional image presented as a baseline contrast in our study. In 1-back condition, the participants decided whether the number shown currently matched the previous number. This condition used more WM load than in 0-back condition. In 2-back condition, the participants decided whether the number shown currently matched the number 2-back in the sequence. This condition used more WM load than in 1-back condition. During WM tasks in the scanner, participants could see the stimuli on an overhead screen and respond by pressing a response pad. We applied block design for the functional imaging study. Each condition is comprised of a single run. Each run contained 3 epochs. Each epoch contained consequent 12 numbers presentation in a 30-second period, followed by fixation on a crosshair in another 30-second period. Participants used a pad near right hand to respond. If the current presented number was the target, they used right index finger to press the pad, if not, used right middle finger. The numbers of correct and incorrect responses were recorded. The task performance was only valid when accuracy rate of 0- and 1-back conditions reached 85% or above.

### Imaging Methods

The 3.0T MR system (Discovery MR750, GE Healthcare) with 8-channel head coil was used to receive brain imaging signals. We applied an established protocol to obtain the functional imaging.^25^ Standard echo-planar imaging method with the protocol of TR = 3000 ms, TE = 35 ms, FA = 90°, FOV = 230 mm^2^, matrix = 64 × 64, 40 slices, slice thickness = 3 mm, and interslice gap = 1 mm, was used for rapid image data acquisition. T1-weighted data were obtained by the protocol of TR = 8.2 ms, TE = 3.2 ms, TI = 450 ms, FA = 12°, FOV = 24O mm^2^, matrix = 256 × 256, 160 slices, and voxel size = 0.9375 × 0.9375 × 1 mm^3^. T2-weighted data were obtained by the protocol of TR = 5700 ms, TE = 100 ms, FOV = 230 mm^2^, matrix = 416 × 416, 20 slices, slice thickness = 5 mm, interslice gap = 2 mm, and number of excitations = 1.

### Data Preprocessing

We followed the protocol in another fMRI study by our group.^25^ In brief, the functional imaging data were preprocessed using Statistical Parametric Mapping 5 (Wellcome Department, University College London, UK) implemented in MATLAB Version 7.9 (MathWorks, Sherborn, MA). The functional images were realigned with the first volume of the series by the method of rigid-body transformation procedure and reslicing to reduce motion artifacts during task performance. After the head motion effect was removed, the volumes were spatially normalized to the Montreal Neurological Institute EPI template. In order to increase the signal-to-noise radio and decreased anatomical variation among participants, 3D Gaussian filter with a full width at half maximum of 6 × 6 × 6 mm^3^ was used to spatially smooth the normalized EPI images.

### Statistical Analysis

Data of the 2 groups, including demographic information, neuropsychological test (MMSE and AD8), and accuracy rate of n-back WM tasks, were compared using 2-tailed *t*-tests or Chi-square tests. For functional dataset analysis, general linear model of hemodynamic response analysis implemented in Statistical Parametric Mapping 5 was employed. To obtain the brain activation patterns for each group, preprocessed scans for all participants were input into the general linear model. Two sets of contrast images were created in each group, including comparison between 0- and 1-back (1 > 0) and 1- and 2-back (2 > 1) conditions. The probability threshold value was set at 0.001 uncorrected with a minimum cluster extent of 3 contiguous voxels. Activation difference maps for 1 > 0-back and 2 > 1-back conditions between Met allele carriers and noncarriers were generated by second-level multiparticipant or between-group random effect analyses. The 1-sample *t*-test was applied for within-group analyses. The 2-sample *t*-test was applied for between-group analyses.

## RESULTS

**FIGURE 1:**
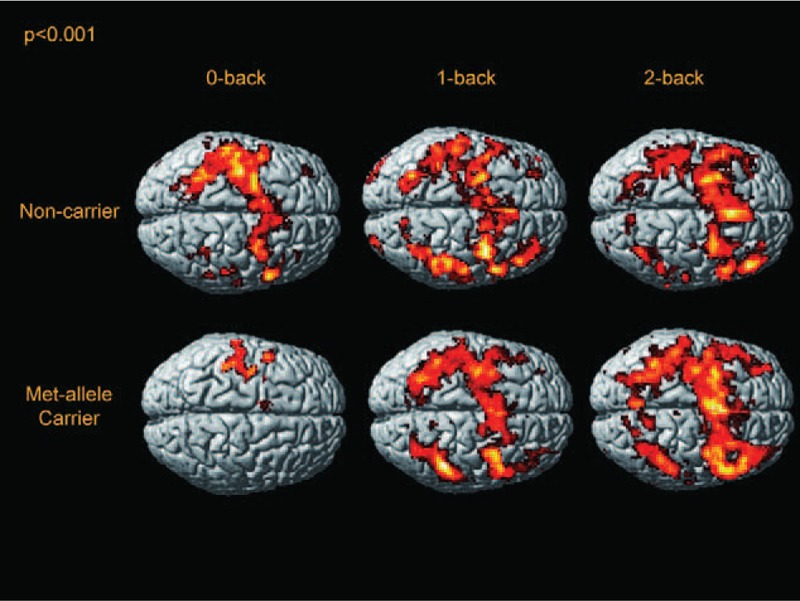
Activation maps of the BDNF Met allele carriers and noncarriers in the n-back task in a surface-rendered projection displayed on a standardized brain atlas (display threshold, *P* < 0.001; extent, 3 voxels). The averages of BOLD signals in 90 seconds of fixation in each single run were used as the contrast. Activation in the bilateral frontal and parietal regions was increased, consistent with the activation of WM circuitry in both groups. In both groups, activation in left frontal region was more prominent than that in the right frontal region, possibly representing right index finger movement during WM tasks. BDNF = brain-derived neurotrophic factor, BOLD = blood oxygenation level dependent, WM = working memory.

**FIGURE 2:**
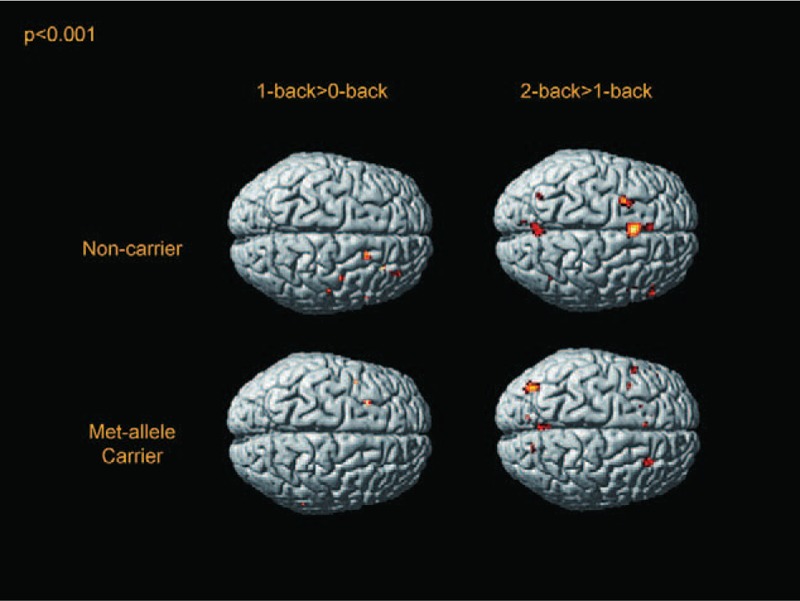
Activation maps of the BDNF Met allele carriers and noncarriers under 1 > 0-back and 2 > 1-back conditions in a surface-rendered projection displayed on a standardized brain atlas (display threshold, *P* < 0.001; extent, 3 voxels). In both groups, brain activation increased in response to each increase in the WM load. Under the high WM load (2 > 1-back), both groups showed further activation in the lateral and medial frontal regions and medial parietal regions bilaterally. BDNF = brain-derived neurotrophic factor, WM = working memory.

**FIGURE 3:**
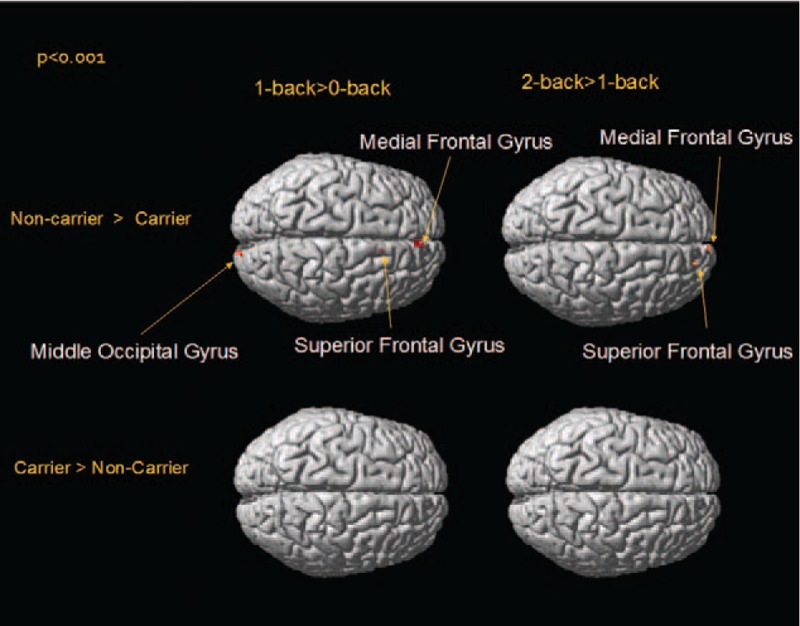
Surface-rendered projections of activation differences between the BDNF Met allele carriers and noncarriers as a function of the WM processing load (1 > 0-back and 2 > 1-back conditions) (display threshold, *P* < 0.001; extent, 3 voxels). This quantitative, voxel-by-voxel comparison of both groups shows lower brain activation in the right middle occipital, superior frontal, and medial frontal gyri in the Met allele carrier group under 1 > 0-back and 2 > 1-back conditions. No increased activation was observed in the Met allele carrier group. BDNF = brain-derived neurotrophic factor, WM = working memory.

Demographic information, neuropsychologic test results, and accuracy rate of WM tasks are shown in Table 1. The 2 study groups did not differ significantly regarding sex, age, MMSE score, AD8 score, and group accuracy rate of WM tasks (*P* *>* 0.05). However, we observed that the accuracy rate of WM tasks tended to decline when WM load increased in both groups. Activation maps of the Met allele carriers and noncarriers during the n-back task are shown in Figure 1. The maps were generated on a standard brain atlas with the probability threshold value set at 0.001 with a minimum cluster extent of 3 voxels. Both groups showed activation in the bilateral frontal and parietal regions, in association with increased WM load, which was consistent with the activation of the WM circuitry. In both groups, activation in the left frontal region was more prominent than that in the right frontal region, possibly representing right index or middle finger movement during task responses. Contrast images comparing between 0- and 1-back (1 > 0) and 1- and 2-back (2 > 1) in each group are shown in Figure 2. Both groups exhibited increased brain activation in response to increase in the WM load, which is consistent with the findings in Figure 1. However, the pattern of increases in brain activation differed between the 2 groups. Under the low WM load (1 > 0-back), the noncarriers showed increased activation in some regions of right frontal lobe, whereas the Met allele carriers showed increased activation in some regions of left frontal and right lateral parietal lobes. Under the high WM load (2 > 1-back), both groups showed further activation in regions of the lateral and medial frontal and the medial parietal regions bilaterally. Activation difference maps for 1 > 0-back and 2 > 1-back conditions between the 2 groups are shown in Figure 3. In both conditions, noncarriers exhibit more brain activation in some brain regions than Met allele carriers do. That is to say, the Met allele carriers exhibit low brain activation in the right middle occipital, right superior frontal, and right medial frontal gyri. The medial frontal gyrus is the medial surface of the superior frontal gyrus (SFG), and the 2 gyri are referred to as the SFG collectively in the subsequent section.

**TABLE 1:**
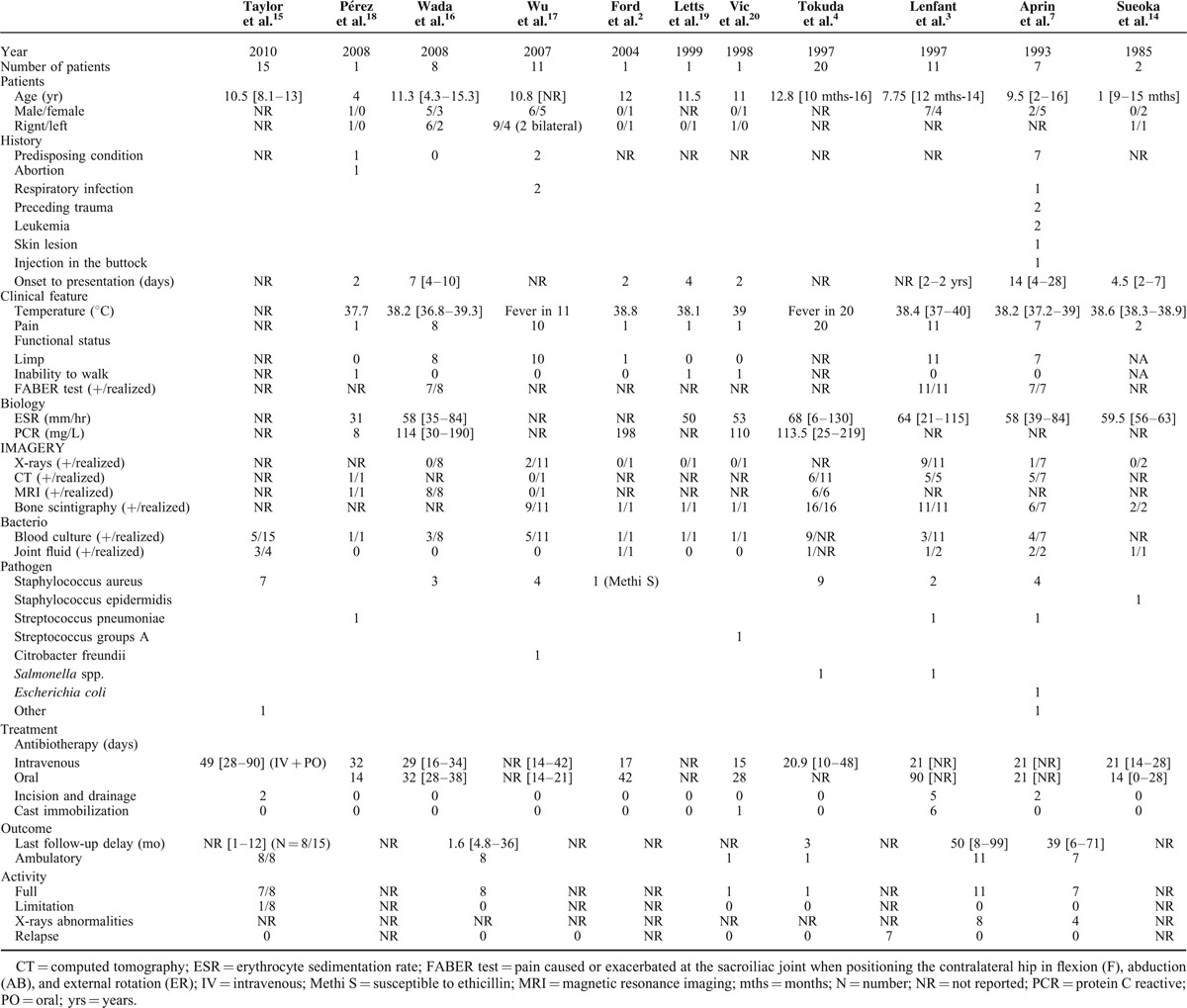
The Demographic Information, Cognitive Function, and Accuracy Rates of n-back WM in the Participants

## DISCUSSION

In the present study, we used a probability threshold value with a high specificity (0.001) to demonstrate different brain activation patterns during n-back WM tasks in BDNF Met allele carriers and noncarriers. Two restricted brain areas with different blood oxygenation level dependent (BOLD)-activation between the Met allele carriers and noncarriers were revealed. When the WM load increased, from 0- to 1-back and from 1- to 2-back, Met allele carriers showed lower brain activation in the midline brain structure, specifically the SFG and middle occipital gyrus (BA18). Brain activation in the dorsolateral prefrontal cortex, essential for coping with increasing brain activity and WM loads,^26^ did not differ significantly between the 2 groups. Increased brain activation was observed in no brain regions among the Met allele carriers.

The role of SFG in WM has been documented in several studies^27–29^ and is responsible for continual update and WM temporal order maintenance.^30^ Patients with left SFG lesions exhibited poor n-back WM performance.^31^ Functional MRI indicated that SFG, particularly spatial WM, is associated with specialized WM functions.^32–34^ In addition, a recent fMRI study demonstrated that SFG is involved in WM for shapes.^35^ Klingberg postulated a superior frontal-intraparietal network implicated in visuospatial WM development.^36^ The role of the occipital visual associate areas (BA18/19) in visuospatial WM has been demonstrated in previous fMRI and PET studies.^32,37–39^ The occipital visual associate areas are part of the proposed dorsal visual pathway specialized in visuospatial information processing; however, its role in numeral WM is unclear.

In our study, low BOLD activation in the SFG and middle occipital gyrus in the BDNF Met allele carriers was observed and can be explained by 2 possible mechanisms. The first is defects in the WM network in BDNF Met allele carriers. In previous animal and human studies, BDNF Met allele carriers typically exhibited poor memory performance.^7–11,40^ If the BDNF Met allele has a negative effect on memory performance, it is rational to assume that low BOLD activation is due to defective development of WM functioning during brain maturation.^41^ The SFG and middle occipital gyrus are considered to be involved specifically in visuospatial WM; therefore, compensatory suppression of these regions might occur for maintaining performance in the visual number-based n-back task in Met allele carriers with WM network defects. However, as mentioned in the Introduction, studies have yielded contradictory results.^14,15^ Efficient use of the intact WM network in BDNF Met allele carriers is another possible explanation for low BOLD activation.

This study has a few limitations. First, serum BDNF levels were not analyzed and correlated to the fMRI results. Although the relationship is controversial, several studies have reported low serum BDNF levels among Met allele carriers.^42–44^ Second, the genetic dose effect of the Met allele on brain activation during WM tasks was not analyzed because of the relatively low number of homozygous Met allele carriers. The genetic dose effect of the BDNF Met allele has been reported by studies examining the hippocampal *N*-acetyl aspartate level by using magnetic resonance spectroscopy and hippocampal activity during episodic memory tasks.^6–15^ Whether the dose effect of the BDNF Met allele exists during WM tasks is unknown. Third, this fMRI study recruited only 11 Met allele carriers and 9 noncarriers among 110 participants, generating a bias and a generalization problem. Additional investigations with a large sample and long-term follow-up for exploring the real cause of low SFG activation in n-back WM tasks are warranted.

In summary, activation patterns seem to differ between BDNF Met allele carriers and noncarriers during WM tasks. A major difference was observed in the SFG and middle occipital gyrus. Further investigation of the mechanism underlying the influence of the BDNF Met allele on the SFG and middle occipital gyrus is warranted.

## References

[1] Tapia-Arancibia L Aliaga E Silhol M New insights into brain BDNF function in normal aging and Alzheimer disease. *Brain Res Rev* 2008; 59:201–220. 18708092 10.1016/j.brainresrev.2008.07.007

[2] Lu B BDNF and activity-dependent synaptic modulation. *Learn Mem* 2003; 10:86–98. 12663747 10.1101/lm.54603 PMC5479144

[3] Hwang IK Yoo K-Y Jung B-K Correlations between neuronal loss, decrease of memory, and decrease expression of brain-derived neurotrophic factor in the gerbil hippocampus during normal aging. *Exp Neurol* 2006; 201:75–83. 16678162 10.1016/j.expneurol.2006.02.129

[4] Dincheva I Glatt CE Lee FS Impact of the BDNF Val66Met polymorphism on cognition: implications for behavioral genetics. *Neuroscientist* 2012; 18:439–451. 22367929 10.1177/1073858411431646 PMC3387519

[5] Hong C-J Liou Y-J Tsai S-J Effects of BDNF polymorphisms on brain function and behavior in health and disease. *Brain Res Bull* 2011; 86:287–297. 21924328 10.1016/j.brainresbull.2011.08.019

[6] Egan MF Kojima M Callicott JH The BDNF val66met polymorphism affects activity-dependent secretion of BDNF and human memory and hippocampal function. *Cell* 2003; 112:257–269. 12553913 10.1016/s0092-8674(03)00035-7

[7] Gong P Zheng A Chen D Effect of BDNF Val66Met polymorphism on digital working memory and spatial localization in a healthy Chinese Han population. *J Mol Neurosci* 2009; 38:250–256. 19424874 10.1007/s12031-009-9205-8

[8] Harris SE Fox H Wright AF The brain-derived neurotrophic factor Val66Met polymorphism is associated with age-related change in reasoning skills. *Mol Psychiatry* 2006; 11:505–513. 16446742 10.1038/sj.mp.4001799

[9] Richter-Schmidinger T Alexopoulos P Horn M Influence of brain-derived neurotrophic-factor and apolipoprotein E genetic variants on hippocampal volume and memory performance in healthy young adults. *J Neural Transm* 2011; 118:249–257. 21190051 10.1007/s00702-010-0539-8

[10] Schofield PR Williams LM Paul RH Disturbances in selective information processing associated with the BDNF Val66Met polymorphism: evidence from cognition, the P300 and fronto-hippocampal systems. *Biol Psychol* 2009; 80:176–188. 18838100 10.1016/j.biopsycho.2008.09.001

[11] Tsai S-J Hong C-J Yu YW-Y Association study of a brain-derived neurotrophic factor (BDNF) Val66Met polymorphism and personality trait and intelligence in healthy young females. *Neuropsychobiology* 2004; 49:13–16. 14730195 10.1159/000075333

[12] Bueller JA Aftab M Sen S BDNF Val66Met allele is associated with reduced hippocampal volume in healthy subjects. *Biol Psychiatry* 2006; 59:812–815. 16442082 10.1016/j.biopsych.2005.09.022

[13] Molendijk ML Bus BAA Spinhoven P A systematic review and meta-analysis on the association between BDNF val(66)met and hippocampal volume – a genuine effect or a winners curse? *Am J Med Genet B Neuropsychiatr Genet* 2012; 159B:731–740. 22815222 10.1002/ajmg.b.32078

[14] Beste C Baune BT Domschke K Paradoxical association of the brain-derived-neurotrophic-factor val66met genotype with response inhibition. *Neuroscience* 2010; 166:178–184. 20034542 10.1016/j.neuroscience.2009.12.022

[15] Huang CC Liu ME Chou KH Effect of BDNF Val66Met polymorphism on regional white matter hyperintensities and cognitive function in elderly males without dementia. *Psychoneuroendocrinology* 2014; 39:94–103. 24275008 10.1016/j.psyneuen.2013.09.027

[16] Hariri AR Goldberg TE Mattay VS Brain-derived neurotrophic factor val66met polymorphism affects human memory-related hippocampal activity and predicts memory performance. *J Neurosci* 2003; 23:6690–6694. 12890761 10.1523/JNEUROSCI.23-17-06690.2003 PMC6740735

[17] Hashimoto R Moriguchi Y Yamashita F Dose-dependent effect of the Val66Met polymorphism of the brain-derived neurotrophic factor gene on memory-related hippocampal activity. *Neurosci Res* 2008; 61:360–367. 18501457 10.1016/j.neures.2008.04.003

[18] Kauppi K Nilsson L-G Adolfsson R Decreased medial temporal lobe activation in BDNF (66)Met allele carriers during memory encoding. *Neuropsychologia* 2013; 51:2462–2468.doi:10.1016/j.neuropsychologia.2012.11.028. 23211991 10.1016/j.neuropsychologia.2012.11.028

[19] Hansell NK James MR Duffy DL Effect of the BDNF V166 M polymorphism on working memory in healthy adolescents. *Genes Brain Behav* 2007; 6:260–268. 16848784 10.1111/j.1601-183X.2006.00254.x

[20] Cerasa A Tongiorgi E Fera F The effects of BDNF Val66Met polymorphism on brain function in controls and patients with multiple sclerosis: an imaging genetic study. *Behav Brain Res* 2010; 207:377–386. 19874854 10.1016/j.bbr.2009.10.022

[21] Galvin JE Roe CM Powlishta KK The AD8: a brief informant interview to detect dementia. *Neurology* 2005; 65:559–564. 16116116 10.1212/01.wnl.0000172958.95282.2a

[22] Folstein MF Folstein SE McHugh PR “Mini-mental state”. A practical method for grading the cognitive state of patients for the clinician. *J Psychiatr Res* 1975; 12:189–198. 1202204 10.1016/0022-3956(75)90026-6

[23] Hong CJ Yu YW Lin CH An association study of a brain-derived neurotrophic factor Val66Met polymorphism and clozapine response of schizophrenic patients. *Neurosci Lett* 2003; 349:206–208. 12951204 10.1016/s0304-3940(03)00828-0

[24] Chen C-J Chen C-C Wu D Effects of the apolipoprotein E ε4 allele on functional MRI during n-back working memory tasks in healthy middle-aged adults. *AJNR Am J Neuroradiol* 2013; 34:1197–1202. 23275593 10.3174/ajnr.A3369 PMC7964576

[25] Chen C-J Wu C-H Liao Y-P Working memory in patients with mild traumatic brain injury: functional MR imaging analysis. *Radiology* 2012; 264:844–851. 22829681 10.1148/radiol.12112154

[26] Owen AM McMillan KM Laird AR N-back working memory paradigm: a meta-analysis of normative functional neuroimaging studies. *Hum Brain Mapp* 2005; 25:46–59. 15846822 10.1002/hbm.20131 PMC6871745

[27] Postle BR Stern CE Rosen BR An fMRI investigation of cortical contributions to spatial and nonspatial visual working memory. *Neuroimage* 2000; 11 (5 Pt 1):409–423. 10806028 10.1006/nimg.2000.0570

[28] Rypma B Prabhakaran V Desmond JE Load-dependent roles of frontal brain regions in the maintenance of working memory. *Neuroimage* 1999; 9:216–226. 9927550 10.1006/nimg.1998.0404

[29] Thomas KM King SW Franzen PL A developmental functional MRI study of spatial working memory. *Neuroimage* 1999; 10 (3 Pt 1):327–338. 10458945 10.1006/nimg.1999.0466

[30] Wager TD Smith EE Neuroimaging studies of working memory: a meta-analysis. *Cogn Affect Behav Neurosci* 2003; 3:255–274. 15040547 10.3758/cabn.3.4.255

[31] Du Boisgueheneuc F Levy R Volle E Functions of the left superior frontal gyrus in humans: a lesion study. *Brain* 2006; 129 (Pt 12):3315–3328. 16984899 10.1093/brain/awl244

[32] Courtney SM Petit L Maisog JM An area specialized for spatial working memory in human frontal cortex. *Science* 1998; 279:1347–1351. 9478894 10.1126/science.279.5355.1347

[33] Haxby JV Petit L Ungerleider LG Distinguishing the functional roles of multiple regions in distributed neural systems for visual working memory. *Neuroimage* 2000; 11 (5 Pt 1):380–391. 10806025 10.1006/nimg.2000.0592

[34] Sala JB Rämä P Courtney SM Functional topography of a distributed neural system for spatial and nonspatial information maintenance in working memory. *Neuropsychologia* 2003; 41:341–356. 12457759 10.1016/s0028-3932(02)00166-5

[35] Yee LTS Roe K Courtney SM Selective involvement of superior frontal cortex during working memory for shapes. *J Neurophysiol* 2010; 103:557–563. 19923241 10.1152/jn.91299.2008 PMC2807238

[36] Klingberg T Development of a superior frontal-intraparietal network for visuo-spatial working memory. *Neuropsychologia* 2006; 44:2171–2177. 16405923 10.1016/j.neuropsychologia.2005.11.019

[37] Carlson S Martinkauppi S Rämä P Distribution of cortical activation during visuospatial n-back tasks as revealed by functional magnetic resonance imaging. *Cereb Cortex* 1998; 8:743–752. 9863701 10.1093/cercor/8.8.743

[38] Jonides J Smith EE Koeppe RA Spatial working memory in humans as revealed by PET. *Nature* 1993; 363:623–625. 8510752 10.1038/363623a0

[39] Smith EE Jonides J Koeppe RA Spatial versus Object Working Memory: PET Investigations. *J Cogn Neurosci* 1995; 7:337–356. 23961865 10.1162/jocn.1995.7.3.337

[40] Yu H Wang Y Pattwell S Variant BDNF Val66Met polymorphism affects extinction of conditioned aversive memory. *J Neurosci* 2009; 29:4056–4064. 19339601 10.1523/JNEUROSCI.5539-08.2009 PMC2668145

[41] Geier CF Garver K Terwilliger R Development of working memory maintenance. *J Neurophysiol* 2009; 101:84–99. 18971297 10.1152/jn.90562.2008 PMC2637004

[42] Bhang S Ahn J-H Choi S-W Brain-derived neurotrophic factor and serotonin transporter gene-linked promoter region genes alter serum levels of brain-derived neurotrophic factor in humans. *J Affect Disord* 2011; 128:299–304. 20674984 10.1016/j.jad.2010.07.008

[43] Elfving B Buttenschøn HN Foldager L Depression, the Val66Met polymorphism, age, and gender influence the serum BDNF level. *J Psychiatr Res* 2012; 46:1118–1125. 22682508 10.1016/j.jpsychires.2012.05.003

[44] Ozan E Okur H Eker C The effect of depression, BDNF gene val66met polymorphism and gender on serum BDNF levels. *Brain Res Bull* 2010; 81:61–65. 19589373 10.1016/j.brainresbull.2009.06.022

